# Magnetic inductive phase shift: a new method to differentiate hemorrhagic stroke from ischemic stroke on rabbit

**DOI:** 10.1186/s12938-017-0354-7

**Published:** 2017-05-30

**Authors:** Qingguang Yan, Gui Jin, Ke Ma, Mingxin Qin, Wei Zhuang, Jian Sun

**Affiliations:** College of Biomedical Engineering, Third Military Medical University, Chongqing, 400030 China

**Keywords:** Magnetic inductive phase shift (MIPS), Hemorrhagic stroke, Ischemic stroke

## Abstract

**Background:**

The major therapy for ischemic stroke is thrombolytic treatment, but severe consequences occur when this method is used to treat hemorrhagic stroke. Currently, computed tomography and magnetic resonance imaging are used to differentiate between two types of stroke, but these two methods are ineffective for pre-hospital care.

**Methods:**

We developed a new brain diagnostic device for rabbits based on electromagnetic induction to non-invasively differentiate two types of stroke. The device includes two coils and a phase difference measurement system that detects the magnetic inductive phase shift (MIPS) value to reflect the tissue’s condition. The hemorrhage model was established through the injection of autologous blood into the internal capsule of a rabbit’s brain. Ischemia was induced in the brain of a rabbit by bilateral carotid artery occlusion. Two types of animal models were measured with our device.

**Results:**

The MIPS value gradually decreased with increasing injected blood and increased with ischemia time. The MIPS changes induced by the two types of strokes were exact opposites, and the absolute values of MIPS variation in the hemorrhagic and the ischemic groups were significantly larger than those of the normal control group (P < 0.05).

**Conclusions:**

The tested technique can differentiate ischemic stroke from hemorrhagic stroke on rabbit brain in a non-invasive, continuous, and bulk monitoring manner by using a simple and inexpensive apparatus.

## Background

Stroke can be classified into two kinds, namely, hemorrhagic and ischemic. The incidence rate of this medical condition continuously increases with the aging of the global population and the intensification of social pressure. The latest statistics issued by the Ministry of Health of the People’s Republic of China reveal that stroke is the first cause of death among all diseases in China, and its standardized mortality rate in the country ranks first in the world. Besides, such a mortality rate continues to increase at a yearly rate of 9%. Approximately 2 million people suffer a stroke each year. Among this population, nearly half die, and roughly 3/4 of the survivors experience varying degrees of disability. Stroke has brought severe economic burdens to China and its people, and this situation emphasizes the urgency for the prevention and control of such a medical condition [1]. Ischemic stroke (obstruction of blood flow) accounts for nearly 80% of all stroke cases, and hemorrhagic stroke (bleeding into brain or on the surface of the brain) accounts for the remaining 20% [2].

The early thrombolytic treatment of ischemic stroke is an established procedure today, but it could be disastrous when performed on patients with hemorrhagic stroke [3–5]. At present, computed tomography (CT) and magnetic resonance imaging (MRI) are used to differentiate hemorrhagic stroke from ischemic stroke. The early use of thrombolytic therapy immediately after the onset of ischemic symptoms could produce satisfactory outcomes [6]. However, such a treatment is not advised to be adopted beyond 4.5 h after the onset of such symptoms because its potential benefits do not outweigh the risk of hemorrhagic complications, which increase with time. The majority of the time after the onset of ischemic symptoms is wasted in transport to and from the diagnostic instruments and in imaging test; thus, only 1–8% of the entire stroke population is given such treatment [7]. This clinical challenge urges the development of new and simple pre-hospital methods that can distinguish between ischemic and hemorrhagic strokes from each other.

Several technologies for distinguishing ischemic from hemorrhagic stroke have been developed over the years. Doppler ultrasonography can accurately identify the states of the cerebral artery (e.g., stenosis, obstruction, convulsion, or ischemia), but it cannot exclude hemorrhagic transformation from the resulting ischemic regions [8]. Electrical impedance tomography (EIT) has been proposed as a possible technology to detect intracerebral hemorrhage (ICH) in an animal model [9] and impedance spectroscopy has been suggested as a method to detect stroke related brain asymmetries in men [10]. However, EIT requires the injection of current through an electrode-skull contact that could reduce the detection precision [11]. Besides, the current is difficult to pass the super-high resistivity skull and may therefore severely reduce the imaging quality [11]. The near-infrared spectroscopy (NIRS) detects hemorrhagic stroke because the hemoglobin absorbs more near-infrared light than other tissues. In a previous study, a hand-held NIRS meter (InfraScan, US) was used to detect a trauma-induced hemorrhage, and 50 hemorrhagic patients were found with a sensitivity of 88% and a specificity of 90.7% [12]. Nonetheless, this method could only detect hematoma at less than 2.5-cm deep below the scalp and with a volume of above 3.5 ml [12]. The same type of meter identified 28 hemorrhage child patients with a sensitivity of 100% and a specificity of 80%, but failed to detect deep or early hemorrhage [13]. The microwave technique depends on the existence of a significant dielectric contrast between blood and other tissues. A previous research adopted two sets of microwave measurement systems and a detection cap containing a microwave patch antenna arrays to detect 45 patients (19 cases of hemorrhage and 26 cases of ischemia) and 65 healthy volunteers [6]. This microwave method could distinguish the difference between hemorrhage and ischemia strokes as well as that between hemorrhage and a healthy condition [6]. However, this technique is extremely sensitive to the shift between the antennas and the hemorrhagic site. Besides, microwave attenuation in the brain is far larger than the magnetic field. Hence, microwave is insensitive to deep hemorrhage.

The MIPS technique is based on the principle of electromagnetic induction and measures the phase perturbation of the induced magnetic field (IMF) to the excitation magnetic field (EMF). The phase perturbation is proportional to the conductivity of the measured object. Changes in conductivity can be detected by the MIPS technique [14]. Hemorrhage and ischemia are both accompanied with changes in the volume and components of intracranial tissues. Given that these changes would later induce changes in the overall brain conductivity, the MIPS technique is able to identify the pathological conditions in the brain. Hemorrhage and ischemia are opposite pathological states. Hemorrhage is the bleeding induced by parenchyma vascular rupture. At the early stage, bleeding reduces the cerebrospinal fluid (CSF). When the compensatory mechanism of CSF ends, the increasing amount of bleeding will increase intracranial pressure (ICP) significantly. Cerebral ischemia is a medical condition in which a certain regionis deprived of oxygen and nutrient-rich blood. Ischemic stroke can be thrombotic type, in which a diseased or damaged cerebral artery becomes blocked or embolic, and the clot (emboli) is formed somewhere other than the brain itself. Ischemia induces abundant neuron necrosis and causes infarct. After long ischemic time, the necrotic tissues can no longer be recovered. The conductivity changes with the type of brain tissue as it decrease from CSF and blood to gray and white matters. Besides, the conductivity changes when the cells are at different pathological states, such as necrosis and dropsy. In this case, the MIPS technique can reflect the tissue conditions. Overseas researches on brain disease detection with MIPS method remain in the level of physical model and simulation experiments. A small number of pilot animal and clinical trials had been conducted by Gonzalez. In 2007 and 2009 [15, 16], Gonzalez used a single exciting coil and a single receiving coil to measure the MIPS changes induced by intraperitoneal injection and cerebral ischemia in rats, and the MIPS changes reflected the volume changes and ischemic degree, respectively. In 2013, he used the same coil structure to examine the breast tissues of five breast cancer patients at four frequencies; the measured results are consistent with the simulated results [17]. Then, he measured the MIPS changes of 46 healthy volunteers and eight patients with CT radiology confirmed brain edema and brain hematoma within 1–200 MHz and used the U test to differentiate between the healthy volunteers and patients and between ICH and brain edema patients at different frequency intervals [18].

Our group has long been engaged in MIPS detection research on cerebral hemorrhage, cerebral ischemia and brain edema, and carried out a large number of animal experiments. The experimental results show that MIPS would gradually reduce as the amount of bleeding increased [19–21]. In this study, a novel coil structure was designed and used to measure the MIPS changes caused by hemorrhage or ischemia on rabbits. In particular, we aimed to distinguish two types of stroke through this simple device. But this is just the feasibility study to explore the usefulness of this concept.

## Methods

### Theory of MIPS technique

**Fig. 1:**
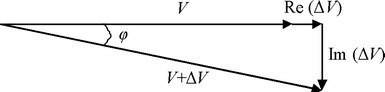
Phasor diagram representing the detected primary signal *V* and secondary signal Δ*V*; the total signal (*V* + Δ*V*) lags the primary signal by an angle *φ*

In MIPS technique, the measured object is always placed between a transmitting and receiving coil, and the current flow in the transmitting coil induces a primary magnetic field. This primary magnetic field then incurs eddy currents in the object that in turn produce a secondary magnetic field. Both the primary and secondary fields are detected by the receiving coil. The primary and secondary signals (*V* and Δ*V*, respectively) can be represented by the phasor diagram shown in Fig. 1. As reported in a previous study [22], if the skin depth of the electromagnetic field in the sample is larger than the thickness of the sample, then Δ*V* is related to *V* as follows:

1 $$ \Delta V/V = Q\omega \mu_{0} \left[ {\omega \varepsilon_{0} \left( {\varepsilon_{r} - 1} \right){-}i\sigma } \right] + R\left( {\mu_{r} - 1} \right), $$

where *ω* is the signal frequency; *σ*, *ε*_*r*_, and *μ*_*r*_ are the electrical conductivity, relative permittivity, and relative permeability of the sample, respectively. *ε*_*0*_ and *μ*_*0*_ are the permittivity and permeability of free space, respectively. *Q* and *R* are geometrical constants. Thus, the total signal (*V* + Δ*V*) detected by the receiving coil lags the primary signal by an angle *φ*, which is approximately proportional to *ω* and *σ*.

### Detection coil

**Fig. 2:**
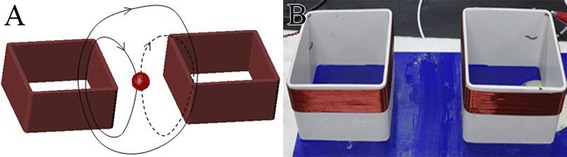
Schematic (**A**) and **B** photograph of the detection coil structure

The MIPS detection coil is illustrated in Fig. 2. Two square-shaped coils (one transmitting coil and one receiving coil) of the same size were used for the experiments. Both coils were constructed from 25 turns of copper wire rolled on a square plastic with a side length of r = 100 mm. The distance between the center of the coils was d = 180 mm. As displayed in Fig. 2A, the transmitting coil produced an EMF (solid line) that passed through the test object (red ball), which in turn generated an IMF (dash line). A part of EMF and IMF was received by the receiving coil. The receiving coil could only receive a small part of EMF because of the large distance between them. The majority of the IMF was received by the receiving coil. The square coils could produce an area between two coils with uniform sensitivity. The resonant frequency of the coil combination at no load condition was determined experimentally to be centered on 14.8 MHz. When the rabbit head was placed in the coil structure, the resonant frequency shifted to 16.4 MHz. When working at this frequency, the magnetic field intensity and MIPS sensitivity were both maximized. Thus, the frequency of 16.4 MHz was selected and used in all experiments.

### Experimental setup

**Fig. 3:**
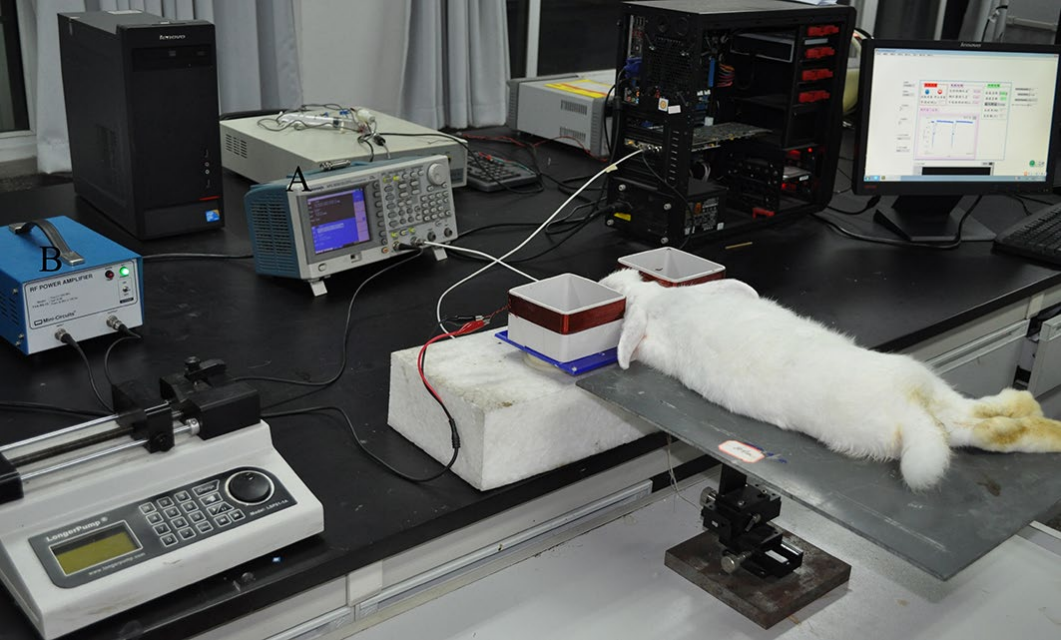
Experimental setup

The experimental setup is shown in Fig. 3. An AFG3252 arbitrary waveform generator (‘A’ in Fig. 3; Tektronix, USA) was used as the signal source. This generator outputted two channels of signals with the same frequency and phase. One signal with an amplitude of 200 mVPP was inputted to an RF instrument power amplifier (‘B’ in Fig. 3, TVA-R5-13, Mini-Circuits, USA). The amplifier had a bandwidth ranging from 0.5 MHz to 1000 MHz, an output power of +35 dBm, and a typical high gain of 38 dB. The output of the amplifier was connected to the transmitting coil. The other signal from the AFG3252 generator with an amplitude of 1 VPP was connected to an input port of a PCI5124 dual-channel high data acquisition card (NI, USA; maximal sample rate = 200 M S^−1^) inserted into the slot of a PC. The output signal from the receiving coil was connected to the other input port of the PCI5124. The rabbits were placed on a polyvinyl chloride plate platform, which could move up–down, left–right, and back–forward. Before measurements, the height and horizontal position of the platform were readjusted, placing the rabbit brain exactly between the two coils. The geometrical position of all subjects was carefully maintained as similar as possible for all measurements. A phase difference measurement program based on a Fast Fourier Transform (FFT) algorithm was compiled on LabVIEW software. This program was used to measure the phase difference between the two input signals to the PCI5124 acquisition card. The sampling rate was set at 100 M/S, and the number of sampling points was 1 million. The changes in the phase were recorded as a function of the ischemia time or injection volume.

### Ethics statement of animal experiments

All experimental protocols were approved by the Animal Experiments and Ethics Committee of the Third Military Medical University, and the care of the animals was performed in accordance with the Declaration of Helsinki and the guidelines issued by the International Association for the Study of Pain [23, 24].

### Experimental animals and grouping

A total of 20 New Zealand rabbits (2.0–2.5 kg in weight) were randomly selected and divided into two groups, i.e., hemorrhage and ischemia groups (each n = 10). To lessen killing and keep consistency, four animals were measured two hours earlier before blood transfusion or ligation in two groups. The data before transfusion or ligation were regarded as control group data. Eight rabbits’ data before transfusion or ligation were randomly selected and regarded as a control group data. All animals were purchased from the Animal Center of Chongqing Daping Hospital and were well attended to before and after the experiment.

### Hemorrhage operations

Considering that ICH mainly occurs in the internal capsule, we established the internal capsule hemorrhage model by injecting self-body blood into the hindlimb of the right capsule. The operation procedure in this study is similar to those in previous research [19–21]. The rabbits were first anesthetized by injecting their ear vein with urethane (25%, 5 ml/kg). For the experimental group, the anesthetic medicine was no longer added after the first anesthesia was completed because the measuring time was not >2 h. For the control group, 3 ml of urethane (25%) was added 2 h after the surgery. Consequently, a longitudinal incision was made on the median of the rabbit head, and the anterior fontanelle and coronal suture were exposed. A hole (d = 1 mm) was drilled 1 mm in front of the coronal suture and 6 mm from the midline. A total of 2 ml of fresh auto blood was extracted from the subcutaneous vein of the hindlimb using a heparinized syringe. A plastic tube (d = 0.7 mm) was introduced to an appropriate depth (H = 13 mm). After surgery, the rabbit was fixed on the platform. The position of the rabbit was readjusted to place its brain exactly in between the two coils (Fig. 3). The measurement system was then connected. With a syringe pump, 1 ml of autologous blood was injected into the internal capsule at a constant speed (1 ml/h). The experimental setup simultaneously measured the MIPS. The data before transfusion were used as the baseline data. After the measurement was performed, 1 ml of 1.5 mol/l KCL solution was injected into the ear vein of the rabbits to execute them.

### Ischemia operations

**Fig. 4:**
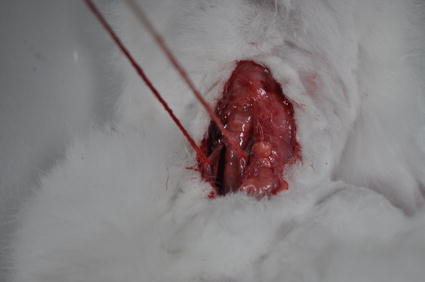
Bilateral common carotid artery in a rabbit

The bilateral carotid artery permanent ligation method was used to establish the ischemia model. The rabbits were first anesthetized by injecting their ear vein with urethane (25%, 5 ml/kg). Under anesthesia, the necks of the rabbits were depilated and disinfected. Subsequently, the middle neck skin was incised and bluntly dissected to expose and separate the bilateral common carotid artery, which was knotted using two 1 mm-thick nylon lines, but not ligated (Fig. 4). After the operation, the rabbit’s head was fixed on the platform and placed in between the two coils. Once the system stabilized, the bilateral carotid artery was tightly ligated, followed by the synchronous measurement of MIPS for 2 h. Consequently, 1 ml of 1.5 mol/l KCL solution was injected into the ear vein of the rabbits to sacrifice them.

### Statistical analyses

Data of the absolute value of MIPS measured in the three groups were presented as mean ± SD. The Wilcox rank sum test was applied to test the difference in the MIPS between the stroke groups and the normal control group in SPSS (SPSS Inc., Chicago, IL, USA). A value of P < 0.05 was considered statistically significant.

## Results

**Fig. 5:**
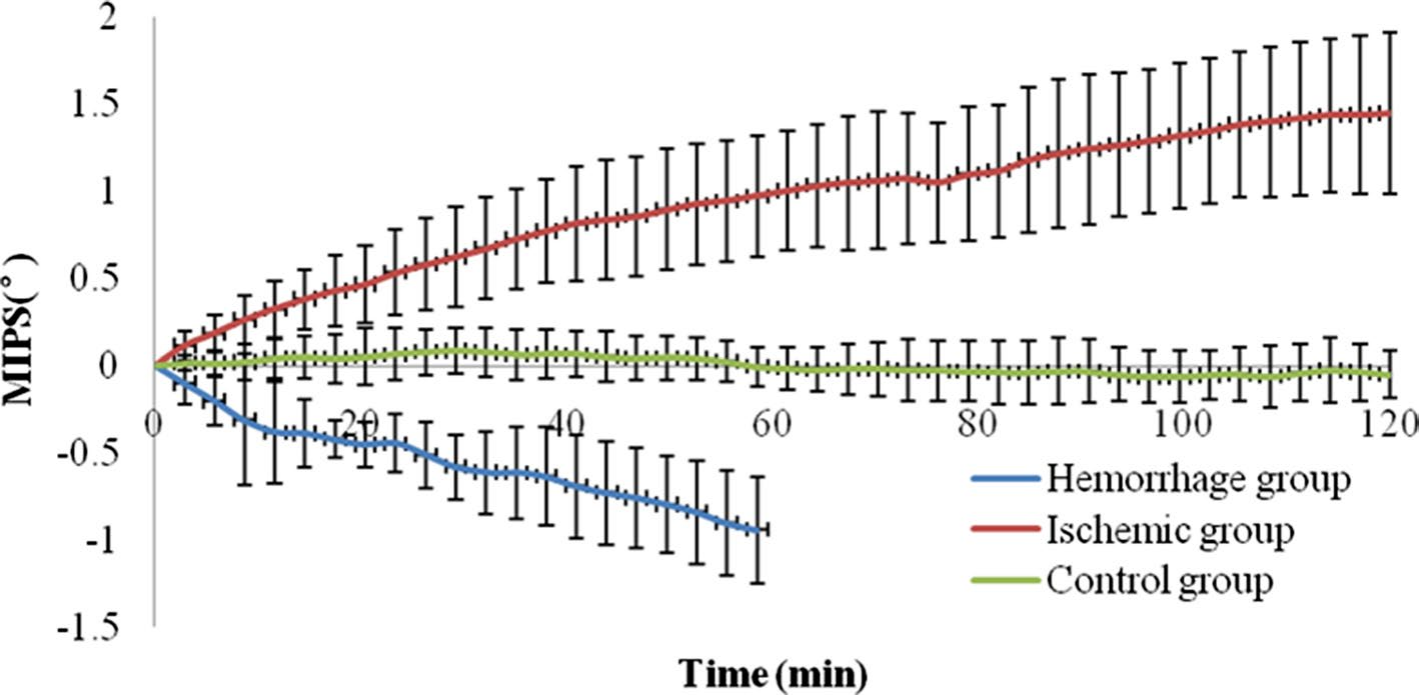
MIPS measurement results for three groups, where the* X-axis* indicates the measurement time, while the* Y-axis* indicates the MIPS data. The data in each group were the average value of all group animals concerned. The data from the hemorrhage group were normalized relative to the baseline data without injection, and the data from ischemic group were normalized to the baseline data before ligation. The data from the control group were also normalized relative to the initial value

The breathing and heart beat signals in each animal data were filtered with wavelet decomposition. For the result of each animal in ischemic and control group, the MIPS data during 2-h were equally separated into 42 successive parts. Then the mean of each part was determined. This processing method is essentially equivalent to obtaining one MIPS data at every 2.85 min. Then the all group animal data were averaged, and standard deviation (SD) was determined. For the result of each animal in hemorrhage group, the MIPS data during 1-h injection were equally separated into 21 successive parts. Then the mean of each part was determined. This processing is essentially equivalent to obtaining one MIPS data at every 2.85 min or every 0.047 ml of injection. Then the all group animal data were averaged. Figure 5 shows the results where the X-axis indicates the measurement time, while the Y-axis indicates the MIPS data. The data in each group were the average value of all group animals concerned. Each animal data from the hemorrhage group were normalized relative to the baseline data without injection. The data from the ischemic groupwere normalized to the baseline data before ligation. The data from the control group were also normalized relative to the initial data. The statistical comparison showed significant difference (P < 0.05) between the any two groups.

## Discussion

A gradual decrease in the MIPS is observed as a function of the injection volume in Fig. 5. After the injection of 1 ml of blood, the average decrease of MIPS is 0.942 ± 0.307°. This result is consistent with our previous simulation and animal experiments [19–21]. At the early stage of hemorrhage, CSF is gradually removed out of the brain. Given that the conductivity of CSF is larger than that of blood, the overall conductivity of the head is decreased as well as the MIPS.

A gradual increase in the MIPS is observed as a function of the ischemic time in Fig. 5. In this case, the average increase for all animals is 1.448 ± 0.467°. This result is consistent with those of the local ischemia measurement in rats conducted by Gonzalez et al. In 2009, Gonzalez et al. used two coaxial coils placed around the rat head to measure the ischemia-caused MIPS change. The subjects were monitored for 24 h. The results showed a significant increase of MIPS as a function of frequency and ischemic time [16]. It has been known for many years that tissue structure exhibits two electrically conducting compartments, the extra- and intracellular spaces, separated by insulating membranes. The conductance of an electric current through such a structure is highly frequency dependent [25]. The biological tissues display a distinct β-dispersion. Thus, measuring the spectral characteristics of the electrical conductivity of biological tissues provides information regarding the structure and composition of such tissues. In 1997, Gersing et al. measured the impedance spectrum of the canine heart muscle and porcine liver with the increase of ischemic time. The results revealed that between 20 and 175 min after the onset of ischemia, the impedance at low frequency increased by about fivefold, but decreased with ischemic time at a frequency higher than 1 MHz [26]. Given that impedance and conductivity are inversely proportional and the working frequency in our experiment was at 16.4 MHz, our results agree with those of Gersing. Gersing et al. explained that in the course of ischemia, the organ tissue exhibits characteristic changes in the impedance spectra mainly because of cell swelling, closing gap junctions, and accumulation of metabolic products that cause the impedance decrease at high frequencies [26].

The variation of MIPS in control group is little with the time. The average MIPS drift (maximum–minimum) in 2 h is 0.333 ± 0.059°, which is significantly less than those in the other two groups (P < 0.05).

Despite the MIPS changing trends for two kinds of stroke were opposite, the amplitudes of variation were small (0.942 ± 0.307° vs 1.448 ± 0.467°), indicating the low sensitivity of the coil system. Therefore, it is necessary to change the coil structure for improving the MIPS sensitivity. Second, all group data show poor consistency. The standard deviation for the ischemic group is particularly large. The reasons are as follows: First, there are large individual differences for all animals. The weight and age have a great impact on the results. Second, it is hard to ensure that each animal was given a sufficient depth of anesthesia. Some rabbits were restless in experiment for poorly anesthetized. Third, it is difficult to maintain that all animal heads were at the same position between the two coils. The head position is of importance, because MIPS sensitivity varies from the different positions between the two coils, which directly affect the measurement results. Fourth, it is also hard to keep the consistency on the injection position and injection depth for all animals. In general, the experimental method should be improved. The positioning device of higher accuracy is required.

In addition to the above problems, animal ICH and ischemia model also should be improved. Although the used auto blood injection model is perfect for our experiment, because its bleeding amount and bleeding rate are controllable for the convenience of comparative studies, its bleeding mechanism differs from the clinical ICH. Another common bleeding model is collagenase-induced bleeding. The bleeding of this model is indeed caused by rupture of blood vessels, but the bleeding amount and rate are uncontrollable. After the initial trial is completed in the future, we will attempt to adopt this model for MIPS experiment. The ischemia model is often created by middle cerebral artery occlusion (MCAO) which conforms to the actuality. However, this model is mostly used for rat and mice. The rabbit has different arterial structure, and the operation is very difficult, so this model is rarely conducted on rabbit for low success rate. Therefore, we used the bilateral carotid artery permanent ligation method to establish the ischemia model on rabbit. Many papers indicate that bilateral carotid artery permanent ligation may only cause mild cerebral ischemia, which may partly explain the low variation of MIPS in our experiment. We will perform this ischemia model test on rat in the future.

## Conclusions

Ischemia in the brain is an important clinical problem that causes serious consequences when treated as hemorrhage. In this study, a novel coil structure based on MIPS technology was tested to distinguish between ischemic stroke and hemorrhagic stroke. Two stroke animal models were used, and the measurements showed that MIPS gradually decreased with the increase of bleeding amount, but it increased with the prolonging of ischemic time. The changing trends of MIPS because of the two types of stroke were completely opposite and the absolute values of variations in MIPS of the hemorrhagic group and the ischemic group were significantly larger than the normal control group. The mechanisms of MIPS changes due to these two types of stroke are different. The analyses given in the previous Sections indicate that the reduction of MIPS at early hemorrhage is caused by the regulating effect of CSF, and the MIPS changes are mainly caused by the volume changes of tissues at early hemorrhage. The ischemia-induced MIPS rise is attributed to the changes of tissue components. At high frequencies, the increase of MIPS is primarily caused by the change of the total ion content in the tissue. With the prolonging of ischemic time, the accumulation of metabolic products in the tissue is increased, which leads to small impedance, but large MIPS. The consistency among all animal results for each group is low mainly because the placement of the rabbit head between the two coils cannot be guaranteed with full consistency. The individual differences (e.g., weight) among the samples used may be another reason for the low consistency. In general, this is just a pilot study to validate the feasibility of this approach to discriminate two types of stroke, and more work need to be performed. Based on the experimental results, the measurement sensitivity for two kinds of stroke is low; the coil system should be improved. Second, the experimental setup also needs to be improved for higher precision. An animal positioning device should be made to maintain the consistency for all animals in the experiment. Besides, work should be done to establish new ICH and ischemia models which are more consistent with clinic.

## Abbreviations

MIPS: magnetic inductive phase shift
EIT: electrical impedance tomography
ICH: intracranial hemorrhage
NIRS: near-infrared spectroscopy
CSF: cerebrospinal fluid
FFT: Fast Fourier Transform
ICP: intracranial pressure
